# Incidence and predictors of morphometric vertebral fractures in patients with ankylosing spondylitis

**DOI:** 10.1186/ar4581

**Published:** 2014-06-16

**Authors:** Kwi Young Kang, In Je Kim, Seung Min Jung, Seung-Ki Kwok, Ji Hyeon Ju, Kyung-Su Park, Yeon Sik Hong, Sung-Hwan Park

**Affiliations:** 1 Division of Rheumatology, Department of Internal medicine, Incheon St. Mary’s Hospital, The Catholic University of Korea, 56 Dongsu-ro, Bupyeong-gu, Incheon 430-720, South Korea; 2 Division of Rheumatology, Department of Internal medicine, College of Medicine, Ewha Womans University, Seoul, South Korea; 3 Division of Rheumatology, Department of Internal medicine, Seoul St. Mary’s Hospital, The Catholic University of Korea, #505, Banpo-Dong, Seocho-Gu, Seoul, South Korea

## Abstract

**Introduction:**

Ankylosing spondylitis (AS) is associated with an increased incidence of vertebral fractures (VFs); however the actual incidence and predictors of morphometric VFs are unknown. The present study examined the incidence and predictors of new VFs in a large AS cohort.

**Methods:**

In total, 298 AS patients who fulfilled the modified New York criteria were enrolled and spinal radiographs were evaluated biennially. Clinical and laboratory data and radiographic progression were assessed according to the Bath AS Disease Activity Index, erythrocyte sedimentation rate, C-reactive protein (CRP), and the Stoke AS spine score (SASSS). VF was defined according to the Genant criteria. The incidence of VFs at 2 and 4 years was evaluated using the Kaplan-Meier method. The age-specific standardized prevalence ratio (SPR) for AS patients in comparison with the general population was calculated.

**Results:**

Of 298 patients, 31 (10.8%) had previous VFs at baseline. A total of 30 new VFs occurred in 26 patients over 4 years. The incidence of morphometric VFs was 4.7% at 2 years and 13.6% at 4 years. Multivariate logistic regression analysis showed that previous VFs at baseline and increased CRP levels at 2 years were predictors of new VFs (odds ratio (OR) =12.8, 95% confidence interval (CI) = 3.6-45.3 and OR = 5.4, 95% CI = 1.4–15.9). The age-specific specific standardized prevalence ratio of morphometric VFs in AS was 3.3 (95% CI 2.1–4.5).

**Conclusions:**

The incidence of morphometric VFs increased in AS. Previous VFs and increased CRP levels predicted future VFs. Further studies are needed to identify the effects of treatment interventions on the prevention of new VFs.

## Introduction

Ankylosing spondylitis (AS) is a chronic inflammatory disease that mainly affects the sacroiliac joint, vertebrae, and spinal ligaments. The disease typically affects male patients and usually becomes apparent between 20 and 30 years of age [1]. Bone is the target in AS and chronic inflammation leads to a wide range of changes, particularly bone remodeling.

Bone complications in AS include new bone formation in the form of syndesmophytes and erosions, generalized osteoporosis, and vertebral fractures (VFs) [2,3]. Earlier studies indicate an increased risk of osteoporosis and morphometric VFs in AS. The prevalence of osteoporosis is 19 to 61% [4] and the estimated prevalence of VFs varies from 9.5% to 32.4% [2,5-8]. These differences may be associated with differences in recruited patients.

The prevalence of VFs is high in AS; however, the diagnosis of VFs is difficult. Only one in three or one in four VFs come to clinical attention, with a typical symptom being back pain [9]. Because back pain is common is AS, misdiagnosis is possible. Therefore, there is a discrepancy between the prevalence of clinical VFs and morphometric VFs. To date, studies comparing the relative risk of VFs among AS patients and the general population have focused on clinical VFs [6,10]. Although many VFs are not diagnosed because the majority of patients suffer only mild back pain, morphometric VFs are associated with a poor quality of life and impaired physical function [11]. Because post-fracture wedging of the vertebrae can contribute to hyperkyphosis and neurologic complications [12], it is important to identify the predictors of morphometric VFs if we are to effectively manage AS patients.

Previous studies report the prevalence of morphometric VFs on x-ray evaluation or fracture vertebral assessment; however, the actual incidence is unknown. One study reported the incidence of VFs, but only investigated clinical VFs [6]. Therefore, the actual incidence of morphometric VFs in AS remains unclear. The objectives of this study were to examine the incidence of morphometric VFs on x-ray evaluation in AS patients and to identify the risk factors associated with new VFs.

## Methods

### Patients

The study enrolled 298 patients (237 men and 61 women) with AS who fulfilled the modified New York criteria for the classification of AS [13] and who presented consecutively between January 2007 and February 2013. This observational cohort study examined pelvis and lumbar spinal radiographs every 2 years to assess structural progression. Patients at two participating centers in South Korea, Seoul Saint Mary’s hospital and Incheon Saint Mary’s hospital, were enrolled between January 2007 and February 2011. The participants’ written consent was obtained according to the Declaration of Helsinki. Exclusion criteria included psoriasis, inflammatory bowel disease, reactive arthritis, thyroid or parathyroid disorders, and chronic renal or liver disease. Patients not evaluated at the time of radiography (±3 months) were excluded. This study was approved by the ethics committee of the Seoul St. Mary’s hospital (XC13RIMI0129K) and the Incheon St. Mary’s Hospital, Catholic University of Korea (XC13RIMI0129O).

### Clinical data

Disease activity at baseline and at 2 years and 4 years was assessed using the Bath AS disease activity index (BASDAI) and laboratory data (erythrocyte sedimentation rate (ESR) and C-reactive protein (CRP) levels, measured every 6 months for 2 or 4 years). Demographic data included age, gender, age at AS diagnosis, disease duration, a history of uveitis, peripheral arthritis, enthesitis, a family history of AS, and the presence of HLA-B27.

### Vertebral fracture assessment

VFs were assessed on lateral radiographs of the lumbar spine (vertebrae T12 to L4) using the standardized semiquantitative method described by Genant *et al*. [14]. The vertebrae were assigned a severity grade based on the visually apparent degree of vertebral height loss. Each vertebra was assigned a grade based on the Genant semiquantitative scale (0 to 3). Grade 0 is normal; grade 1 (mild) represents a reduction in vertebral height of 20 to 25%; grade 2 (moderate) represents a reduction of 25 to 40%; and grade 3 (severe) represents a reduction of over 40%. VFs were defined as a reduction in vertebral height ≥20% [14]. Grading was performed by two experienced investigators (KYK and IJK). There were few discrepancies and the two investigators reached a consensus. The inter-observer variability was calculated using Cohen’s kappa value (kappa = 0.81). If a 2-year radiograph was missing and the 4-year radiograph showed no evidence of fracture, it was assumed that there was no fracture at 2 years.

### Radiographic scoring

Radiographs of the lumbar spine and pelvis were obtained at baseline and after 2 years and 4 years of follow up. Sacroiliitis was assessed by viewing images of the sacroiliac joint and was graded according to the New York criteria [13]. The reader was blinded to the clinical details of the patients. Radiographic changes related to AS in the lumbar spine were assessed using the Stokes ankylosing spondylitis spine score (SASSS). To obtain the SASSS, the anterior and posterior vertebrae of the lumbar (T12 lower to S1 upper) spinal segments were scored (0 to 3 points each, where 0 = normal, 1 = erosion, sclerosis, or squaring; 2 = syndesmophyte formation; and 3 = a bridging syndesmophyte) [15]. A change ≥2 SASSS units in 2 years was defined as significant; such patients were placed in the SASSS progression group.

### Treatment

We also investigated medications such as non-steroidal anti-inflammatory drugs (NSAIDs), sulfasalazine, methotrexate (MTX), tumor necrosis factor (TNF) inhibitors, calcium, and bisphosphonate. All patients treated with TNF inhibitors received the drug throughout the study period. Patients who were on other medications for more than 50% of the study period were considered sustained users. For patients treated with TNF inhibitors, baseline radiographs were performed at the beginning of TNF inhibitor therapy.

### Statistical analysis

Statistical analyses were performed using PASW statistics 18. Data were expressed as the mean ± SD. Normally distributed demographic and radiologic variables were compared using the independent *t*-test and non-normally distributed variables were compared using the Mann-Whitney *U*-test. The Chi-squared test was used to compare categorical variables. Multivariate logistic regression analysis was performed to investigate the association between variables and the incidence of new VFs. All variables with a *P*-value <0.10 in the univariate analysis were incorporated as explanatory variables. The cumulative incidence of new VFs over time was estimated using the Kaplan-Meier method. The log-rank test was used to compare the incidence rate according to different variables.

When comparing the prevalence of radiographic morphometric VFs in AS patients with that in the Korean general population [16], all AS patients under 40 years of age were excluded because all members of the general population group were ≥40 years old. We applied the age-specific prevalence of morphometric VF in the Korean general population study to the age distribution of our AS cohort, and calculated an age-standardized prevalence ratio (SPR) and its 95% CI. All tests were two tailed and a *P*-value <0.05 was considered statistically significant.

## Results

Table 1 shows the baseline characteristics of the 298 AS patients. The mean age of the patients was 33 ± 11 years, 79.5% were male, and 92.1% were HLA-B27-positive. The mean disease duration was 3.8 ± 5.1 years and the mean age at diagnosis was 30 ± 11 years. The mean BASDAI score was 5.3 ± 1.8, the mean grade of sacroiliitis was 3.1 ± 0.7, and the mean SASSS score was 8.6 ± 15.9. The mean baseline ESR was 28.6 ± 25.8 mm/h and the mean CRP was 1.7 ± 3.1 mg/L. Of the 298 patients included in the study, 118 (39.6%) received TNF inhibitors. The clinical and radiological parameters and treatments are shown in Table 1.

**Table 1:** Baseline characteristics of 298 patients with ankylosing spondylitis

**Characteristics**	**Number (%)**	**Mean ± SD**
Sex		
Women	61 (20.5)	
Male	237 (79.5)	
Age (years)		33.9 ± 10.9
Age at diagnosis (years)		30.1 ± 10.8
Disease duration (years)		3.8 ± 5.1
Family history of AS	27 (9.1)	
HLA-B27-positive^1^	244 (92.1)	
Back pain	293 (98.7)	
History of peripheral arthritis	190 (63.8)	
History of enthesitis	71 (23.8)	
History of uveitis	87 (29.7)	
BASDAI, score		5.4 ± 1.8
Sacroiliitis grade		
Right		3.1 ± 0.7
Left		3.1 ± 0.7
Mean sacroiliitis grade		3.1 ± 0.7
SASSS, score (range)		8.6 ± 15.9 (0 to 69)
ESR, mm/h		28.6 ± 25.8
CRP, mg/L		1.7 ± 3.1
Patients on NSAIDs	245 (82.2)	
Patients on sulfasalazine	141 (47.3)	
Patients on MTX	86 (29.1)	
Patients on TNF inhibitors	118 (39.6)	
Patients on calcium	22 (11.1)	
Patients on bisphosphonate	8 (2.7)	

^1^Confirmed in 265 patients. AS, ankylosing spondylitis; BASDAI, Bath AS disease activity index; SASSS, Stokes ankylosing spondylitis spine score; NSAIDs, non-steroidal anti-inflammatory drugs; MTX, methotrexate; TNF, tumor necrosis factor.

### Vertebral fractures

**Figure 1:**
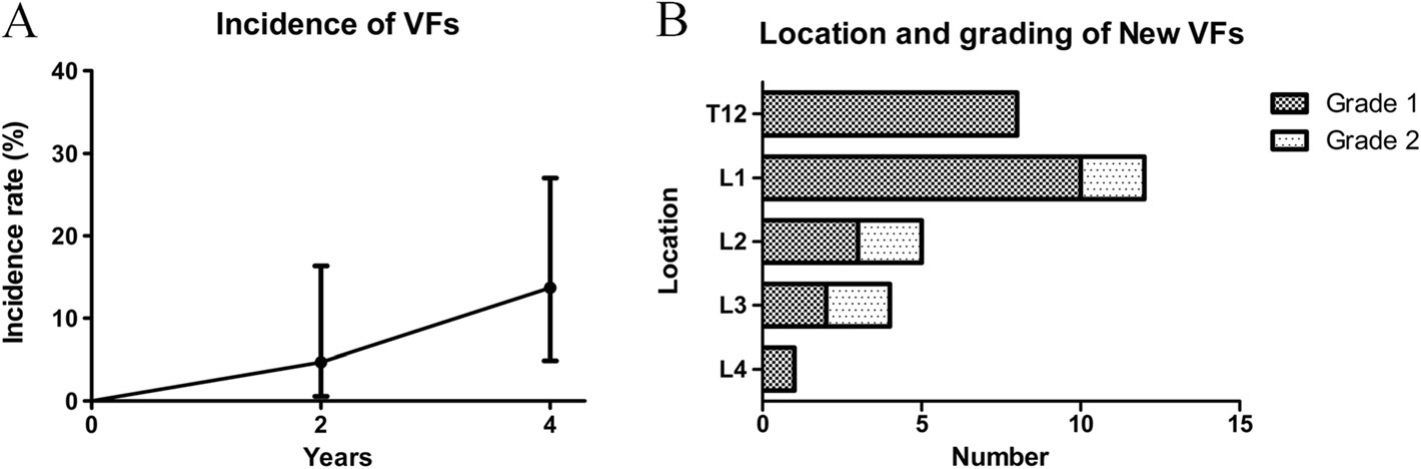
**Incidence of vertebral fractures (VFs) over 4 years.** Thirty VFs of 298 patients were observed for a total of 852 person-years over the study period. **(A)** The incidence of VFs was 4.7% at 2 years and 13.6% at 4 years. **(B)** This resulted in a total of 30 fractures, most commonly at vertebra L1: 24 VFs were Genant grade 1 and 6 VFs were grade 2. Error bars represent the mean (SD).

Of the 298 patients, 31 (10.8%) had previous VFs at baseline. The 298 patients were observed for a total of 852 person-years over the study period. Follow-up radiographic data were available for 287 (96.3%) patients at 2 years and for 131 (40.6%) patients at 4 years. Thirty new VFs were identified in 26 (8.7%) patients. Figure 1A shows that the incidence of VFs was 4.7% at 2 years and 13.6% at 4 years. The most common location of a new VF was L1 (40%). Twenty-three patients had one VF, two had two VFs, and one had three VFs. In total, 24 grade-1 fractures and 6 grade-2 fractures were detected; no grade-3 fractures were found (Figure 1B). Nine patients among the 26 patients with new VFs experienced SASSS progression over the first 2 years. In six out of nine patients, the incident vertebral fractures occurred at same location as SASSS progression.

### Comparison of the characteristics of patients with and without incident VFs

A comparison of the characteristics of patients with and without new VFs revealed that those with new VFs were less likely to be HLA-B27-positive or to have uveitis (*P* = 0.083 and 0.012, respectively) as shown in Table 2. Significant increases in the SASSS score and CRP levels over the first 2 years were more common in AS patients with new VFs than in those without (*P* = 0.026 and 0.020, respectively). The prevalence of previous VFs at baseline was significantly higher in patients with new VFs than in those without (*P* <0.001).

**Table 2:** Comparison of characteristics of patients with and without new vertebral fractures

**Variables**	**Patients without new VFs**	**Patients with new VFs**	***P*-value**
**Mean ± SD or number (%)**	**(n = 272)**	**(n = 26)**	
Male	215 (79)	22 (85)	0.617
Age, years	33.8 ± 10.9	35.2 ± 12.2	0.526
Age at diagnosis, years	30.0 ± 10.6	31.6 ± 11.9	0.459
Disease duration, years	3.8 ± 5.1	3.6 ± 5.2	0.847
Family history of AS	24 (9)	3 (12)	0.717
HLA-B27-positive^1^	226 (93)	18 (82)	0.083
Back pain	267 (98.5)	26 (100)	1.000
History of peripheral arthritis	176 (65)	14 (54)	0.290
History of enthesitis	62 (23)	9 (35)	0.226
History of uveitis	85 (32)	2 (8)	0.012
Presence of baseline VF	22 (8)	9 (35)	<0.001
Baseline BASDAI score	5.5 ± 1.8	5.2 ± 1.7	0.726
Baseline mean sacroiliitis grade	3.1 ± 0.7	3.1 ± 0.9	0.945
Baseline SASSS score	15.8 ± 1.0	13.1 ± 17.8	0.138
Significant SASSS progression^2^	47 (18)	9 (39)	0.026
Baseline ESR, mm/h	26.2 ± 1.6	24.4 ± 20.6	0.397
Baseline CRP, mg/L	1.8 ± 3.2	1.0 ± 1.3	0.251
Increase in ESR at 2 years	153 (56)	12 (46)	0.542
Increase in CRP at 2 years	50 (22)	9 (47)	0.020
Patients on NSAIDs	223 (82)	22 (85)	1.000
Patients on sulfasalazine	124 (46)	12 (46)	1.000
Patients on MTX	82 (30)	4 (15)	0.119
Patients on TNF inhibitors	112 (41)	6 (23)	0.093
Patients on calcium	31 (12)	2 (8)	0.751
Patients on bisphosphonate	8 (3)	0 (0)	0.475

^1^Confirmed in 265 patients; ^2^change ≥2 SASSS units over the first 2 years post-baseline. VF, vertebral fracture; AS, ankylosing spondylitis; ESR, erythrocyte sedimentation rate; CRP, C-reactive protein; BASDAI, Bath AS disease activity index; SASSS, Stokes ankylosing spondylitis spine score; NSAIDs, Non-steroidal anti-inflammatory drugs; MTX, Methotrexate; TNF, tumor necrosis factor.

**Figure 2:**
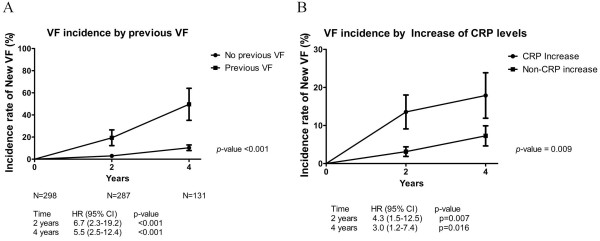
**Comparisons of incidence of morphometric vertebral fractures (VFs). (A)** The risk of VF was significantly increased in patients with previous VFs at baseline compared with those without. **(B)** The risk of occurrence of new VFs at 2 years and 4 years was higher in patients with an increase in C-reactive protein (CRP) levels at 2 years post-baseline than in patients with a reduction.

The use of TNF inhibitors was more common in patients without new VFs than in those with new VFs; however, the difference was not statistically significant. The number of patients treated with NSAIDs, DMARDs, or calcium and bisphosphonates did not differ between the two groups.Figure 2 shows the difference of the incidence of new VFs according to the previous VFs at baseline and the increase in CRP levels at 2 years post-baseline. When the patients had a previous VF at baseline, the hazards ratio (HR) for VF incidence increased to 6.7 (95% CI = 2.3 to 19.2) at 2 years and to 5.5 (95% CI = 2.5 to 12.4) at 4 years. Additionally, when the CRP level at 2 years was higher than that at baseline, the HR for VF incidence increased to 4.3 (95% CI = 1.5 to 12.5) at 2 years and to 3.0 (95% CI = 1.2 to 7.4) at 4 years.

### Risk factors associated with new VF incidence

Multivariate logistic regression analysis showed that the incidence of new VFs was independently associated with previous VFs at baseline and with increased CRP levels at 2 years, with odds ratios (OR) of 12.8 (95% CI 3.6 to 45.3) and 5.4 (95% CI 1.4 to 15.9), respectively (Table 3). Subgroup analysis showed that the increase in CRP levels was independently associated with the development of new VFs in patients with no previous VFs at baseline (OR = 4.8, 95% CI = 1.4 to 15.9).

**Table 3:** Multivariate analysis of risk factors associated with new vertebral fractures

**Variable**	**Total AS patients**			**AS patients without baseline VFs**		
	**(n = 298)**			**(n = 268)**		
	**Odds ratio**	**95% CI**	***P*-value**	**Odds ratio**	**95% CI**	***P*-value**
TNF inhibitor						
Baseline VF	12.8	3.6 to 45.3	<0.001			
HLA-B27						
Uveitis						
SASSS progression						
CRP increase	5.4	1.7 to 17.7	0.005	4.8	1.4 to 15.9	0.011

AS, ankylosing spondylitis; TNF, tumor necrosis factor; SASSS, Stokes ankylosing spondylitis spine score; CRP, C-reactive protein.

Table 4 shows the age-specific SPR for morphometric VFs in AS patients compared with the morphometric VFs expected in the Korean general population [16]. There was a significant increase in the risk of morphometric VFs among AS patients aged over 40 years (standardized Prevalence rate (SPR) = 3.3, 95% CI = 2.06 to 4.54).

**Table 4:** Age-specific prevalence ratio for vertebral fractures in ankylosing spondylitis patients

**Age (years)**	**Age ≥40 years**	**General population**	**SPR (95% CI)**
	**(n = 108)**	**(n = 2684)**^**1**^	
	**Number of VF/total (%)**	**Number of VF/total (%)**	
<40		Not available	
40 to 49	14/69 (33.3%)	26/494 (5.2%)	
50 to 59	7/28 (25.0%)	70/840 (8.3%)	
60 to 69	3/7 (28.6%)	177/964 (18.4%)	
70 to 79	3/4 (75%)	91/386 (30.8%)	
Total	27/108 (25%)	364/2684 (13.6%)	3.3 (2.1 to 4.5)

^1^Data from Shin *et al*. [16]. VF, vertebral fracture; SPR, standardized prevalence ratio.

## Discussion

This is a first study to evaluate the incidence of morphometric VFs in AS patients and to identify the risk factors associated with new VFs. The results show that AS patients have an increased risk ratio of morphometric VFs compared with the general population. In this study, using radiographs, the incidence of morphometric VFs was estimated to be 4.7% at 2 years and 13.6% at 4 years. The risk of morphometric VFs was about three times higher than that in the general population. Previous studies report an increased prevalence of clinical VFs in AS patients compared with the general population, which is consistent with the results reported herein [6,10].

The increased risk for VF in AS patients is due to biomechanical changes in the material and structural components of the spine and to osteoimmunology [17]. Radiographic progression, such as syndesmophyte formation and vertebral bridging, is associated with a reduction in spinal flexibility. This increased rigidity means that a simple fall or other minor trauma can result in VFs [18]. Another explanation for the increased risk of VFs in AS is low bone mineral density (BMD). Reduced BMD is found in patients with early disease, independently of spinal mobility and exercise levels [19]. Bone loss is associated with VFs in AS patients [5,7]. It is possible that early trabecular bone loss in the spine increases the risk of fracture, and that heterogeneous bone stiffness increases the risk [17]. Both rheumatoid arthritis (RA) and AS are associated with low BMD and fractures [20].

To date, several studies have examined the prevalence of morphometric VF in AS and the associated risk factors. Known risk factors include male gender, age, low BMD, disease duration, high levels of disease activity, and peripheral joint involvement [2,3,5-7,17,21]. However, until now, no studies have identified factors that predict the incidence of new VFs in AS patients. The present study shows for the first time that previous VFs and elevated CRP levels are risk factors for occurrence of new VFs.

Only 30% of VFs receive clinical attention. Despite this, patients with a VF are at increased risk of a future fracture [22]. Once an initial VF is sustained, the risk of a subsequent VF increases significantly. This phenomenon has been termed the vertebral fracture cascade. The mechanisms underlying this fracture cascade are not fully understood, creating uncertainty regarding the prevention of further fractures [23]. In patients with postmenopausal osteoporosis, the strongest predictor of a future VF is a prior VF. Gehlach *et al*. showed that previous VFs were associated with a 7.3-fold increase in the risk of a subsequent VF [24]. In agreement with this, the results of the present study show that prior VFs are associated with an increased incidence of VFs in AS patients. It is important to know whether the strong relationship between a previous VF and a new VF is due to BMD or to the abnormal spinal biomechanics associated with the prior fracture; however, this was not clarified in the present study. A further large prospective cohort study will be designed to investigate this.

The results of the present study also suggest that appropriate anti-inflammatory therapy may prevent the incidence of new VFs in AS patients with and without previous VFs. In the case of RA (the most common type of chronic inflammatory arthritis), CRP levels are associated with VFs [25]. Previous studies also show that a reduction in CRP levels is correlated with increased BMD in both the hip and lumbar spine in AS patients [26,27]. It is possible that the increase in CRP may lead to a reduction in BMD, thereby increasing the risk of VF. Also CRP levels are associated with disease activity in AS [28,29]. An increase in CRP levels may be associated with the aggravation of back pain and stiffness, leading to increased rigidity. This, in turn, may cause new VFs. Recent prospective cohort studies show that CRP is an independent risk factor for VF in the elderly general population [30,31], and Ishii *et al*. suggested that composite strength indices show an inverse relationship with CRP levels [32]. Taken together, CRP levels may predict the incidence of VF in AS patients and in the elderly general population. Fracture prevention is indicated in high-risk AS patients with a previous history of VFs or with active inflammatory conditions.

In a previous study, radiographic damage has been associated with VF in AS. Vosse *et al*. reported that modified (m)SASSS correlated with mean wedging at the thoracic spine and hyperkyphosis [33]. In the current study, univariate analysis revealed an association between SASSS progression and the incidence of new VF (OR: 2.9, 95% CI = 1.19 to 7.09; data not shown); however, this did not reach statistical significance in multivariate analysis. However, we must point out that Vosse *et al*. performed a cross-sectional study whereas we performed a longitudinal study. Therefore, the relationship between the observed radiographic damage and the prevalence and incidence of VF may be different. Furthermore, we only examined the incidence of VF at T12 to L4. Vosse *et al*. found no association between mSASSS and VF in the lumbar spine. Further large prospective studies, including both the thoracic and lumbar spine, are needed to validate the relationship between radiographic damage and the incidence of new VF.

The medical treatment of AS has improved dramatically over the last few decades. TNF inhibitors markedly reduce symptoms such as back pain and enthesitis [1,34,35]; however, it is still unclear whether these new treatments lead to a reduction in bone complications. Here, we report the effects of TNF inhibitors on the incidence of morphometric VFs in AS patients. Univariate analysis showed that treatment with TNF inhibitors led to a moderate reduction in the incidence of VF; however, multivariate analysis did not identify a protective effect. Because our results suggest that increased inflammation is associated with the risk of VF, the inhibition of inflammation through the administration of TNF inhibitors has a possibility to prevent new VFs; therefore, the effects of TNF inhibitors should be studied in a future randomized control study of AS patients with a high inflammatory status. Vosse *et al*. report that NSAIDs reduce the risk of clinical fracture in AS patients [10]. Continuous use of NSAIDs reduces syndesmophyte formation and prevents progressive rigidity of the spine, making patients less vulnerable to VF [36]. The effects of NSAIDs on fracture repair have been demonstrated in mouse models [37]; however, we did not find that NSAIDS prevented new VF.

Cooper *et al*. reported that female AS patients aged ≥50 years showed a 7.6-fold increase in the risk for VF when compared with the general population [6]. A recent large retrospective cohort study compared the risk of clinical VFs in AS patients with that in healthy controls. AS patients had an increased risk of clinical VFs, with an OR of 3.28 (95% CI = 1.51 to 7.02) [10]. The SPR of morphometric VF risk identified in the present study is similar to that of clinical VF.

This study has several limitations. First, we were not able to investigate the relationship between smoking and the risk of VF. Smoking is a risk factor for spinal radiographic progression [38] and is associated with VF in AS patients [7]. Unfortunately, we did not examine smoking habits in this study due to insufficient information at baseline. Second, we only assessed fractures from T12 to L4. Because AS patients also show an increased incidence of thoracic VFs, we may have underestimated the total incidence in our study population.

## Conclusions

We found that the incidence of morphometric VFs in AS patients was 4.7% at 2 years and 13.6% at 4 years, and that previous VFs and elevated CRP levels are predictors of future VFs. Further studies are needed to identify the effects of treatment interventions on the prevention of new VFs.

## Abbreviations

AS: ankylosing spondylitis; BASDAI: Bath ankylosing spondylitis disease activity index; BMD: bone mineral density; CRP: C-reactive protein; ESR: erythrocyte sedimentation rate; HR: hazards ratio; MTX: methotrexate; NSAID: non-steroidal anti-inflammatory drug; RA: rheumatoid arthritis; SASSS: Stoke ankylosing spondylitis spine score; SPR: specific standardized prevalence ratio; TNF: tumor necrosis factor; VF: vertebral fracture.

## Competing interests

The authors have no competing interests to declare.

## Authors’ contributions

KYK contributed to statistical analysis, interpretation of data, and drafting of the article. IJK contributed to conception, design, and analysis of data. SMJ contributed to conception, design, and acquisition of data. SKK contributed to conception, design, and acquisition of data. JHJ contributed to conception, design, acquisition of data, and helped to draft the manuscript. KSP contributed to conception, design, and acquisition of data. YSH and SHP contributed to design, acquisition of data, analysis and interpretation of data, and revising of the article. All authors read and approved the final manuscript.
